# Sample preparation for actinide isotopic analysis using FIB-SEM

**DOI:** 10.1007/s10967-025-10667-1

**Published:** 2025-12-23

**Authors:** A. D. Wood, S. A. Dunn, P. Kaye, M. Higginson, J. Robb, R. W. Harrison

**Affiliations:** 1 Department of Mechanical and Aerospace Engineering, School of Engineering, University of Manchester, Oxford Road, Manchester, England; 2 AWE Nuclear Security Technologies, Aldermaston, Reading, RG7 4PR England

**Keywords:** Actinide analysis, Mass spectroscopy, Nuclear forensics, Radiochemistry, Focussed ion beam

## Abstract

Capabilities to extract and analyse nanogram-scale uranium dioxide (UO_2_) samples to allow spatial isotopic analysis were established by the development of techniques from wider literature. A Helios 5CX Focussed Ion Beam Scanning Electron Microscope (FIB-SEM) adapted for alpha-emitting radioactive material was used to extract samples of 2–80 ng from a depleted UO_2_ pellet. These were analysed by Thermal Ionisation Mass Spectrometry (TIMS) and Triple Quadrupole Inductively Coupled Mass Spectrometry (ICP-QQQ-MS). TIMS produced measurements for ^234^U, ^235^U, ^236^U, and ^238^U weight-percent (wt%) with relative standard uncertainties (RSU) of between 0.26 and 11%. ICP-QQQ-MS measured the ^235^U/^238^U ratio with an RSU of 37.4%.

## Introduction

Actinide analysis is a key component of nuclear security and the safe operation of the nuclear fuel cycle, making the development of safer and more efficient analytical processes essential [1]. The reduction of sample size is a common goal across radioanalytical fields due to the chemo- and radiotoxic nature of their associated materials: actinides, lanthanides, and decay products, for example [1, 2]. Such samples impose a number of complications on any task in order to protect both workers and the surrounding environment [3–6]. These may include working conditions like radiation shielding and dose-allowances for workers, as well as facility limitations on material mass, activity, isotopic composition, and the volume/form of waste effluent. Further, the challenge of low analyte quantity may spring from these constraints or, alternatively, from limited available material as can be the case in analysis applications such as nuclear forensics [7]. These complications can be reduced by shrinking the mass, and therefore activity, of samples. Smaller samples do, however, carry their own challenges such as more difficult preparation, a decrease in the measurable quantity of material, and a less representative description of a heterogeneous bulk.

FIB-SEMs can image and manipulate material at the scale of hundreds of nanometres, allowing the precise extraction of micron-sized samples from bulk material. This gives control over the area from which the sample is taken, reduces the risk of contamination, and only wastes the material that must necessarily be removed for the sample to be freed. A FIB provides the capability to mill away surface material by sputtering with high-energy ions, deposit layers of material by the decomposition of an injected precursor gas, and produce images by the collection of secondary electrons or ions [8]. This is combined with an SEM in a dual-beam system, allowing simultaneous non-destructive imaging as well as further electron microscopy analysis techniques such as Energy Dispersive Spectroscopy (EDS) and Electron Backscatter Diffraction (EBSD) for chemical and crystallographic fingerprinting [9]. Sub-micron analysis and milling precision can be used complementarily to enhance the selectivity of sampling: material can be taken exclusively from specific features, phases, or regions of interest. This control of sample content enables high spatial resolution that may be lost in bulk analytical techniques.

This work looks at the production of micron-scale samples (or “lift-outs”) using FIB-SEM and their subsequent analysis by various mass spectrometry techniques. Triple Quadrupole Inductively Coupled Plasma—Mass Spectrometry (ICP-QQQ-MS) and Thermal Ionisation Mass Spectrometry (TIMS) were applied to measure the uranium isotopes of bulk and lifted-out samples, probing measurement quality and any isotopic or material heterogeneity. The purpose of this work was to develop the method for lift-out extraction and analysis (e.g. sample size and mass, preparation, dissolution methods, technique sensitivity) on a majority single-phase, homogeneous sample material; future work on more complex materials and properties (such as trace chemical impurities) may then be carried out with the benefit of a more robust foundation from this method development.

## Experimental

### Materials

Chemically pure grade nitric acid 68% (VWR, France) was used for dissolution and separations. For the ion exchange separation of uranium, UTEVA Resin (Triskem International, FR) was used.

For uranium reference materials, a concentration standard (998(3) mg L^− 1^ U) (Fisher Chemical), and certified isotopic standards CRM 145 (0.7111(4) wt% ^235^U) and CRM U005a (0.5000(3) wt% ^235^U) (NBL, USA), were used.

Depleted UO_2_ powder (0.298 ± 0.002 wt% ^235^U) from the United Kingdom National Nuclear Laboratory (UKNNL) was analysed in this work. It was produced from fresh uranium via the UK Integrated Dry Route (IDR) [10]. The powder was densified by taking 1 g and cold-pressing it into a green body pellet before being sintered in a tube furnace for 5 h at 1700 °C in a 5% H_2_ atmosphere. This resulted in a 96.0(5) % dense pellet, as determined by the Archimedes method.

In preparation for microscopy, the pellet was cut vertically in half on a slow-speed circular saw. One half was set in resin, then mechanically polished to 0.25 µm [11]. Following this, it was gently heated in a furnace to soften the non-conductive resin, allowing the polished half to be removed and mounted onto an aluminium SEM stub with Silver DAG.

### Methods

A Thermo Scientific Helios 5CX DualBeam gallium FIB-SEM system was used to prepare the micro-samples from bulk materials. As the instrument was intended for work with alpha-active materials (uranium, plutonium, and mixed oxides), a number of adaptations were made to accommodate the complexities of radioactive samples: a retractable sputter shield, an interfaced argon glovebox, and vent-line filters. For large-scale milling, the sputter shield can be inserted to cover the electron beam (e-beam) polepiece to prevent sputtered material from being redeposited into its sensitive workings. The argon glovebox provides a sealed environment in which samples can be packaged or unpackaged, preventing their direct exposure to the wider lab. This prevents material contamination of the wider-lab while also protecting the sample from moisture, oxygen, and environmental contamination. A gate valve, through which a loading arm transfers samples, connects the sealed environments of the glovebox and FIB chamber. Specialised Ion Getter Pumps (IGP) were required to allow the FIB to remove the glovebox’s argon from its chamber. They still have difficulty pumping argon, however, so repeated nitrogen purging is still necessary to preserve pump lifetime and ensure complete argon-removal. High Efficiency Particulate Air (HEPA) filters were installed on the vent lines of both the Helios and glovebox to contain any loose material that it extracted by the pumps. A Gas Injection System (GIS) deposits layers of platinum to create welds and protective barriers during sample preparation. Often used in tandem with a GIS, a tungsten nanomanipulator needle can physically handle samples within the chamber.

Although well established, the conventional in-situ lift-out production method will be included here for reference as all samples produced for this work generally followed the same steps [12]. The location should be chosen such that the lift-out volume is as homogeneous as possible; thus, the lift-out will be most representative of the phase or feature from which it is taken. Varying milling rates will also add complexity as supervision and re-milling over certain areas may be required. In addition, boundaries/pores will reduce the material mass of the lift-out and create weak-points on an already delicate sample. To produce a lift-out, a layer of platinum (usually between 250 nm and 1 µm thick) is first deposited over the desired area to reduce unintentional damage of the extracted material. Following this, trenches will be milled either side of the platinum layer, exposing the volume of material to be extracted. A further, smaller, trench can be milled at one end to allow the micromanipulator needle easier access to the lift-out. A “J-cut” (so called as the milling patterns resemble the letter “J”) is then made, separating the lift-out almost entirely from the bulk with a small bridge of material to hold it in place. The micromanipulator needle is carefully lowered and positioned, then welded to the lift-out with additional platinum. Secured to the micromanipulator, the final bridge of material can be cut, freeing the lift-out from the pellet. It is then moved to a copper TEM grid (or similar holder), welded into place, then separated from the micromanipulator with a final cut.

The ICP-QQQ-MS system used was an Agilent 8900: a triple quadrupole tandem mass spectrometer (ICP-MS/MS) with a collision reaction cell (CRC) to reduce polyatomic interferences. During analysis, the reaction chamber was run in O_2_ mode: the first quadrupole (pre-CRC) filtering for the analyte ions and the second (post-CRC) for the analyte plus O_2_. Chemical, process, and instrument blanks were run in addition to sample solutions to account for contamination. A rhodium internal standard is run continuously to account for matrix effects and instrument drift, uranium concentration standards for instrument non-linearity, and CRM 145 for mass bias correction.

Solid UO_2_ samples were prepared for analysis by dissolution in 0.5 mL of 6 M nitric acid, followed by aliquoting and dilution with deionised (DI) water to produce 10 mL analyte samples of the required U concentration. In addition to lift-out samples, bulk sample solutions of 1 ppm and 5 ppb U were prepared to provide points of comparison.

The uranium in each lift-out sample solution was isolated by UTEVA Resin ion exchange. To prepare the solutions, they were acidified to 6 M with additional 68% nitric. Ion exchange columns were loaded with UTEVA Resin, then conditioned with 6 M nitric acid. Samples were added to the columns 1 mL at a time and allowed to sit before being released. Columns were rinsed with further 6 M nitric acid before the uranium was eluted with 0.01 M nitric acid, added 1 mL at a time and allowed to sit before releasing. The eluted U solution was acidified with additional 68% nitric for 5 mL of 0.3 M for ICP-QQQ-MS analysis.

ICP-QQQ-MS isotope count rates for ^234^U, ^235^U, ^236^U, and ^238^U were used to calculate isotopic ratios of the uranium in the sample. Instrument non-linearity and mass bias were corrected following the approach suggested by Graczyk et al. [13].

TIMS measurements were conducted on a Thermoscientific Triton. A peak jumping method was employed with beam decay corrections applied during the measurement. The CRM U005a was run alongside the samples to allow for mass bias corrections to be made. Sample composition in ^234^U, ^235^U, ^236^U, and ^238^U weight-percent was measured.

Samples were dissolved in 50% nitric acid for an approximately 1 ppm U solution. A 1 µL aliquot of solution was taken and placed onto a carburised zone-refined rhenium filament, where it was then dried at 1.5 A to fix the material to the filament surface. An expanded uncertainty was applied to the measurement, taking into account the 3 major contributions to the measurement: the uncertainty associated with the certified values of CRM U005a, the standard deviation of the repeat measurement, and the uncertainty from the determination of the isotopic ratio for each measurement.

## Results and discussion

### Method development

#### ICP-QQQ-MS uranium isotopic detection limits

**Fig. 1:**
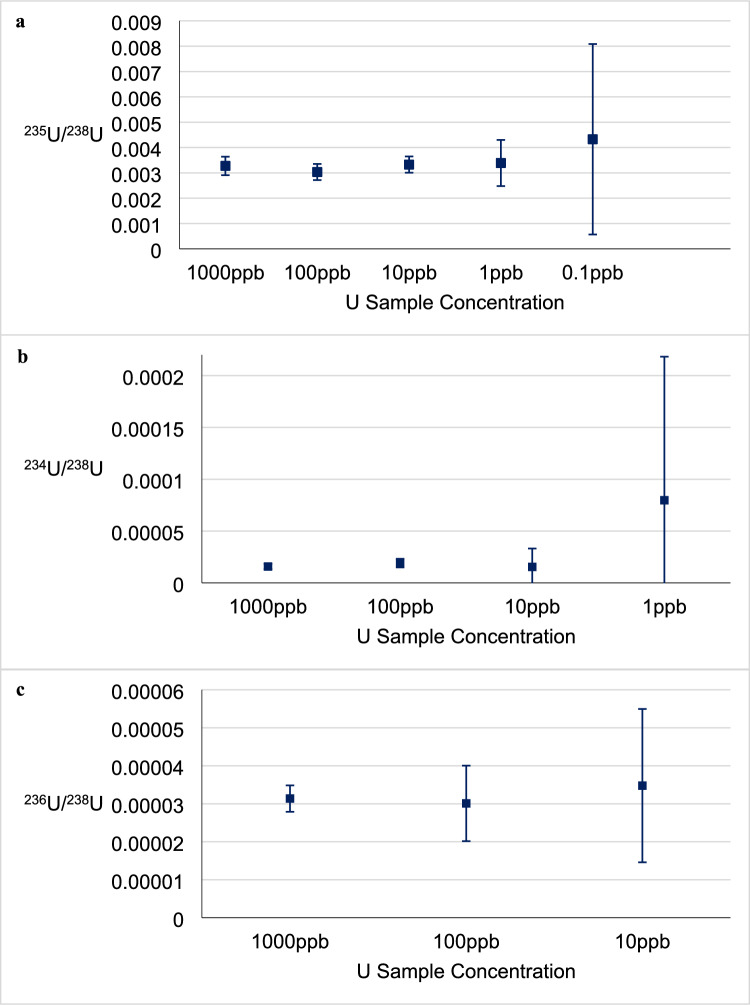
ICP-QQQ major U isotope results of samples with known concentrations (1000 ppb, 100 ppb, 10 ppb, 1 ppb, and 0.1 ppb) plotted with standard uncertainties (*k* = 1). Only samples for which counts were detected are plotted. **a** ^235^U/^238^U atomic ratios, **b** ^234^U/^238^U atomic ratios, and **c** ^236^U/^238^U atomic ratios

To determine the minimum uranium concentration and therefore lift-out size required for major isotopic analysis on the ICP-QQQ-MS, a range of test samples from the U concentration standard were created and measured. Using the provided U concentration of 998.3 ± 3.1 mg L^− 1^ and solution density of 1.011 ± 0.005 g cm^− 3^ at 20 ºC, the concentration of U was found to be 987.4 ± 5.8 µg g^− 1^ or parts-per-million (ppm). The standard was gravimetrically diluted to produce samples of 1000, 100, 10, 1, and 0.1 ng g^− 1^ or parts-per-billion (ppb). These samples of known concentrations were then measured for ^234^U, ^235^U, ^236^U, and ^238^U on the ICP-QQQ-MS to observe the quality of data provided. Figure 1 shows that below 10 ppb U, ^234^U and ^235^U measurement uncertainty increased considerably while ^236^U dropped below the limit of detection. The target minimum concentration for lift-out sample solutions was therefore set at 10 ppb U.

#### Lift-out preparation for ICP-QQQ-MS and TIMS

One of the more common uses of FIB-SEM is the production of Transmission Electron Microscope (TEM) lamellae [14]. Due to milling rates, the typical size of a sample prepared by Ga FIB is in the range of 10–20 µm length, 10 µm depth, and 1–2 µm width (pre-polishing) [15]. Such samples will have a volume of around 300 µm^3^, equating to roughly 3 ng of UO_2_. Reilly et al. [16] have shown that around 5 ng is sufficient to obtain some major U isotopic measurements using TIMS and Quadrupole (Q)-ICP-MS, though for this work it was found that larger masses were needed to achieve the necessary U concentrations for ICP-QQQ-MS. For this reason, standard lift-out sizes were used for the TIMS measurements, but larger samples (approximately 100 µm length, 12 µm depth, and 6 µm width) of around 65 ng were produced for ICP-QQQ-MS (discussed in later section “Large lift-out preparation for ICP-QQQ-MS”). As they were not intended for TEM use and maximising material is desirable, extraction followed the same method as TEM lamellae but stopped before any thinning or polishing.

Three lift-outs, each of an estimated 2.0(4) ng, were produced for TIMS analysis. Before further sample preparation, the grids were first observed under an optical microscope to ensure that the lift-outs were still attached. Grid and sample were then dissolved in preparation for analysis.

At this size, a small increase in sample dimensions will result in a large relative increase in volume, with only a very minimal impact on the milling time. For instance, a width of 3 or 4 µm would provide a respective 50 and 100% increase in volume.

#### Large lift-out preparation for ICP-QQQ-MS

**Fig. 2:**
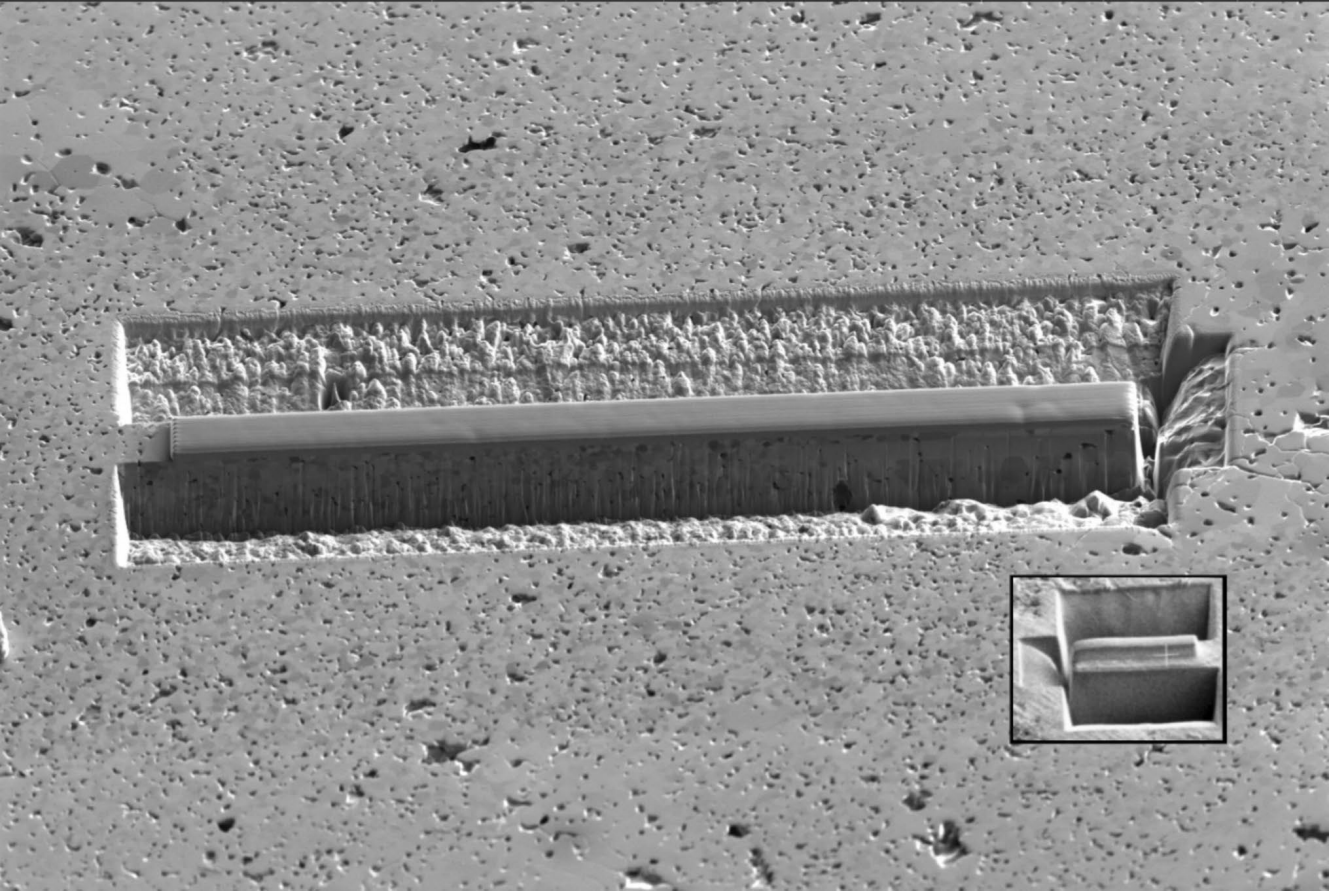
Ion beam secondary electron image of a large lift-out before it has been extracted from the surface of a pellet, with a to-scale inset image of a 15 µm length sample for size comparison

The method used for larger lift-outs was essentially a scaled-up version of the smaller lamellae, as shown in Fig. 2.

Aside from the longer milling times, the main issue with a larger lift-out is that it is more difficult to tell when the beam has fully cut through the material; a deeper cut leads to more redeposition inside the milling area (unless the cut is widened, taking more time) and less signal escaping, making images less clear. Higher currents can be used to speed up the process, but this brings greater redeposition and increased sample damage by both gallium implantation and amorphization [17]. The use of plasma- (typically utilising a collimated beam of xenon plasma) or laser- (a femtosecond laser incorporated into a FIB-SEM) FIB could be more appropriate for samples of this size; future work must be carried out comparing the efficacy of these techniques, as well as how each affects the sample surface and lift-out itself, to determine which is best suited for ICP-QQQ-MS samples [18, 19].

**Fig. 3:**
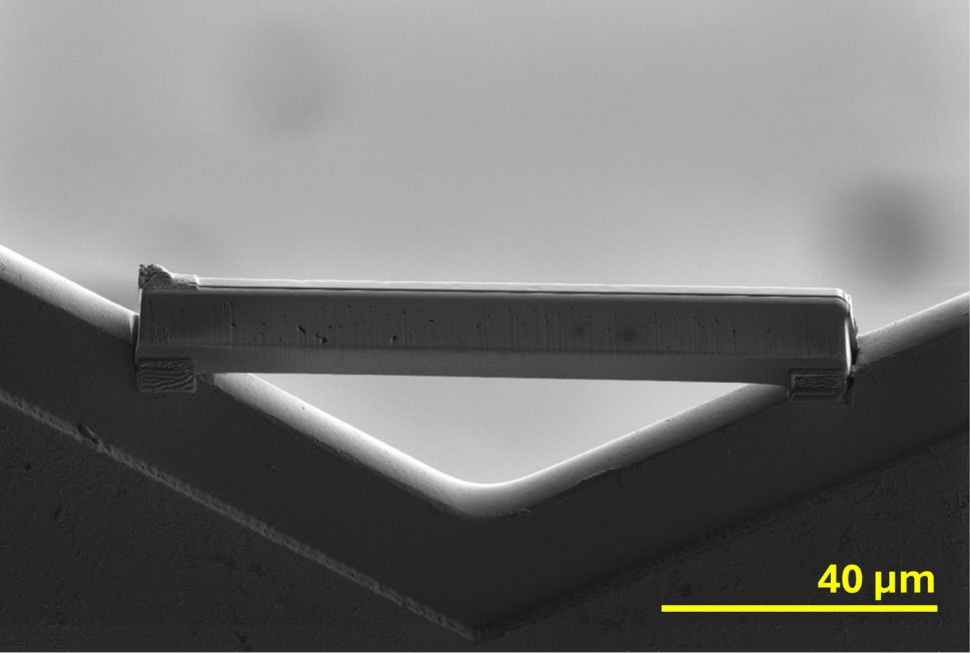
SE Ion Beam image of a large lift-out once it has been extracted from a pellet and secured to a copper TEM grid. The grid has been milled to provide spaces in which both ends of the lift-out can sit

In the case of larger lift-outs, the grid may need to be machined to some extent with the ion beam to provide a more secure space for the sample to be fixed (as seen in Fig. 3).

3 larger lift-outs were extracted for ICP-QQQ-MS. The target mass for these samples was 100 ng, equating to lift-out dimensions of approximately 100 µm length, 5 µm width, and 20 µm depth. The intention was, following dissolution and dilution, to have 10 mL of 10 ppb U solution for each sample. The lift-outs (copper grids still attached) were each dissolved in 0.50(2) mL of 6 M nitric acid, then diluted up to 10.00(6) mL by the addition of DI water. Estimating the size of the lift-outs from SEM images actually placed them at 62(6), 65(6), and 72(6) ng. As a result, the theoretical concentration of uranium in the solutions were in the range of 6–8 µg L^− 1^ (roughly 6–8 ppb); falling short of the previously established target.

The mass-disparity was a consequence of underestimating the amount of material that would be lost from the sample during its removal from the bulk. The cuts made to separate the bottom of the lift-out from the pellet are, necessarily, angled as the rest of the pellet will block the beam at a 90 º incidence. Additionally, the cuts are made at perhaps one third of the lift-out’s height so that the angled beam will emerge from the opposing face, making it possible to monitor the progress with the electron beam and determine when the full width has been cut along the entire length. The consequences are firstly that the bottom third of the lift-out will be left behind, and secondly that the extracted lift-out will be wedge-shaped between the entry of the ion beam on one side and its exit on the other (visible in Fig. 3). The result is a substantial drop in its volume as it is extracted from the surface. To account for this in future work, initial trenches should be of a depth at least 1.5 times greater than the desired height of the lift-out.

Reducing the volume of solution could have increased the uranium concentration, though 10 mL proved necessary in this case for running subsequent analysis.

#### Separation chemistry/Grid materials

The use of a TEM grid, glass needle, or some other form of holder is necessary for tracking and manipulating the microscopic lift-outs. Copper TEM grids (approx. 3 × 1.5 x 0.3 mm) were used in this study, with the entire grid being dissolved alongside the uranium sample during sample preparation as it would not have been possible to remove the lift-out by hand. A rough estimate places the added mass of copper in the vicinity of 12 mg which, when compared with at most 80 ng of uranium, represents an overwhelming majority in the solution. TIMS measurements did not appear to indicate any issues stemming from this, but the ICP-QQQ-MS would need a large reduction in Cu concentration to prevent an increase to instrument background and drop in sensitivity. It was decided to isolate the uranium from the solution by ion exchange with UTEVA Resin, then conduct measurements on the purified uranium solutions.

When comparing the results of the solutions with separated lift-out uranium (nominally still 6–8 ppb) and the calibration standards run alongside them, it became apparent that the concentration in UO_2_ lift-out samples was closer to 1 ppb U (discussed later in “Isotopic Analysis Results”). Though it is possible that this difference was a result of other sample preparation stages, it most likely stems from the recovery in the separation process. There were several opportunities in which uranium may have been lost, such as solution transfer between containers and uranium not captured, or subsequently released, by the resin. In addition, contamination was observed through the measurement a process blank. This could possibly have come from the fume hood that the separation was performed in, cross-contamination between the samples themselves, or from the wider laboratory as it is not a designated cleanroom [20].

To cut out the extra preparation step, future work will be carried out using gold TEM grids which should not dissolve when submerged in nitric acid. These can be removed once the lift-out has dissolved, avoiding solution contamination with large quantities of metals. By minimising the number of processes, the opportunities for additional contamination and sample loss are lessened. Reilly et al. [16] make the same point against the use of copper grids: recommending glass needles to avoid the need for separations.

Once unseparated samples have been analysed, it would be interesting to return to a uranium separation with a yield-tracer like ^233^U to properly investigate the effect that it has on the process as a whole. It should be noted that the impact of this process will be largely reduced, or missing entirely, where separations capabilities are mature.

### Isotopic analysis results

#### TIMS

**Fig. 4:**
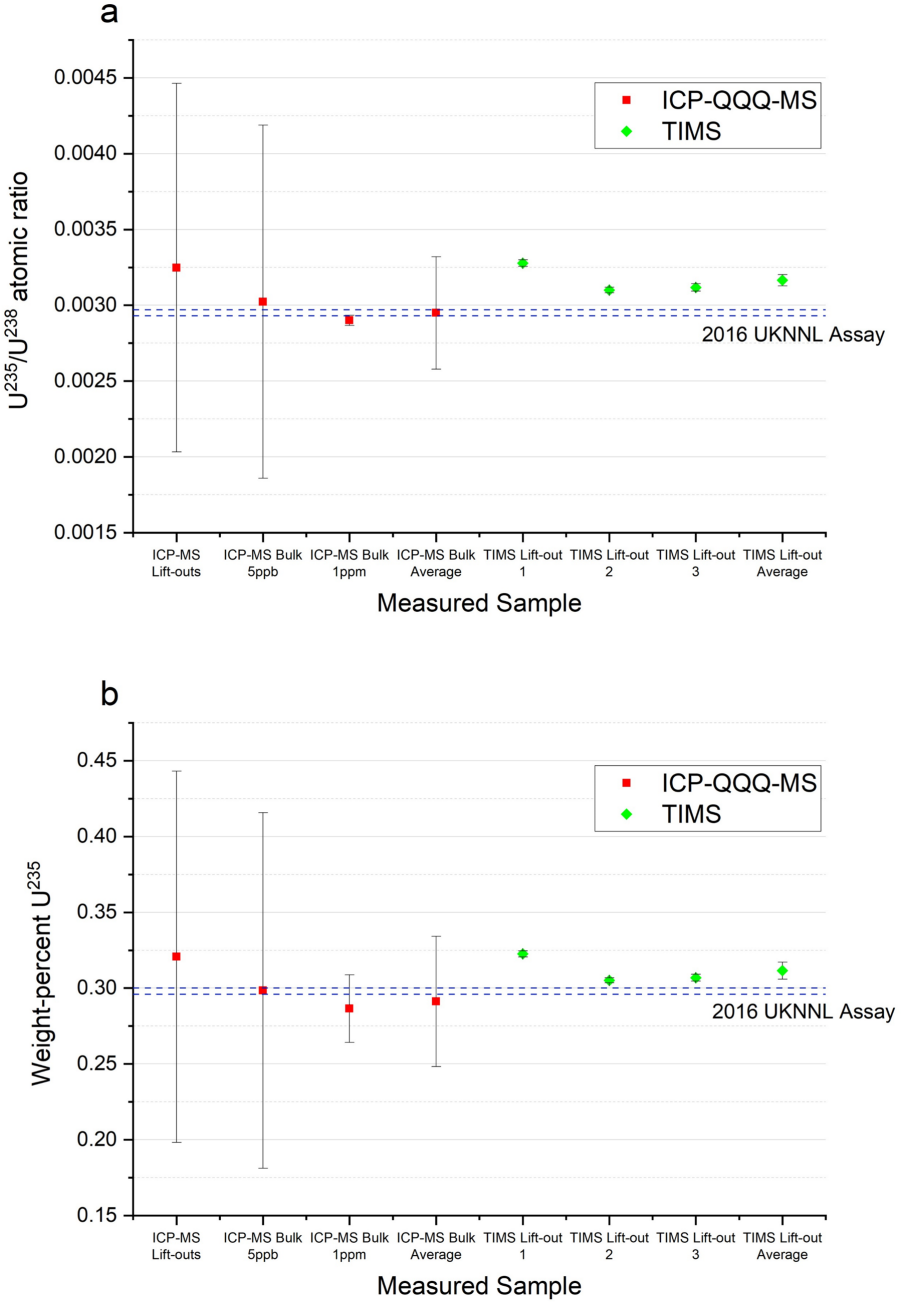
ICP-QQQ-MS and TIMS ^235^U isotopic measurements of UO_2_ material, plotted with standard uncertainties (*k* = 1) and UKNNL assay upper and lower limits as dashed lines. **a** ICP-QQQ-MS ^235^U/^238^U atomic ratio corrected for non-linearity and mass bias, and calculated TIMS ratios. **b** ICP-QQQ-MS ^235^U wt% calculated from measured atomic ratio using the UKNNL assay values for ^234^U and ^236^U, with TIMS ^235^U wt% also included for comparison

The isotopic composition in weight-percent of the uranium in the three lift-out samples, as measured by TIMS, is provided in Table 1. This is presented alongside the values provided by the UKNNL material assay, allowing some comparison to be drawn. The ^235^U wt% and ^235^U/^238^U atomic ratios are also plotted with the ICP-QQQ-MS results in Fig. 4. Generally, the TIMS isotope measurements are higher than the assay values. When the coverage factor is expanded to *k* = 2, however, the majority fall into agreement. The ^234^U and ^236^U measurements for lift-out 2 and ^235^U for lift-out 1 all vary significantly from the other samples. Interestingly, an older assay measurement from 1998 reports 0.312 ± 0.007 wt% ^235^U; this value agrees well with the average TIMS measurement of 0.312 ± 0.006 wt% ^235^U, as well as encompassing the ^235^U wt% measurements of lift-outs 2 and 3 in its wider uncertainty range. The lower assay value used in Table 1 (0.298 ± 0.002 wt% ^235^U) had been chosen as it was more recent (2016) and appeared in three measurements, where the 1998 measurement was only reported once. Lift-outs’ 2 and 3 weight-percent ^235^U were closer to the 2016 assay value than lift-out 1, though they are even more consistent with a single 2011 assay result of 0.300 ± 0.002 wt% ^235^U. One possible explanation for the variance and consistency in reported and measured results is that there is some isotopic heterogeneity within the material: the result will vary, therefore, depending on the location from which the sample is taken. This could be investigated by the extraction of further FIB-SEM lift-outs from a variety of locations within the material, followed by TIMS analysis to produce a spatially-resolved isotopic composition. Additionally, a technique with high spatial resolution, such as nanoscale Secondary Ion Mass Spectrometry (nanoSIMS), could be used to probe any variation in the enrichment of the material [21].

**Table 1:** TIMS major U isotope measurements (weight percent) for each lift-out, their combined average, and the values provided by NNL’s material assay. Combined standard uncertainty (*k* = 1) provided where possible

	^234^U (wt%)	^235^U (wt%)	^236^U (wt%)	^238^U (wt%)
Lift-out 1	1.78(12)E-3	0.3226(19)	5.6(9)E-4	99.7(3)
Lift-out 2	2.83(16)E-3	0.3051(16)	1.74(19)E-3	99.7(3)
Lift-out 3	1.50(11)E-3	0.3068(22)	2.4(3)E-4	99.7(3)
Lift-out Average	2.0(4)E-3	0.312(6)	8.5(43)E-4	99.7(5)
Assay	1.3E-3	0.298(2)	< 2E-4	99.695(2)

#### ICP-QQQ-MS

Uranium concentrations in lift-out solutions were found to be substantially lower than expected at 0.993 ± 0.043 ppb, 3.77 ± 0.25 ppb, and 0.792 ± 0.066 ppb ^238^U. To a lesser degree, the 5 ppb bulk solutions were also of lower-than-expected concentrations, between roughly 2.5 and 3 ppb U. The consequence of this was that, of the major isotopes planned, counts were detected only for ^235^U and ^238^U. The ^235^U/^238^U ratio, therefore, was the only ratio that could be determined, though even this had large uncertainty from counting statistics. The measured ratio of ^235^U/^238^U found from the lift-out solution was 3.2 ± 1.2 × 10^− 3^, while the 5 ppb solution gave 3.0 ± 1.2 × 10^− 3^, and 1 ppm gave 2.901 ± 0.0034 × 10^− 3^. Figure 4a shows how these values compare to the assay ^235^U/^238^U ratio of 2.950 ± 0.020 × 10^− 3^. There still appears to be visible instrument non-linearity as the ratio trends downwards with increasing concentration (and so counts), possibly due to the low count-rates of the standards that were being used to correct it.

For both lift-out and 5 ppb solutions, there were similar variations in the ^235^U/^238^U ratios between repeat samples. Table 2 provides a side-by-side of the ICP-QQQ-MS measurements for both lift-outs and 5 ppb U solutions before any additional processing. The sample number simply provides the running order of both samples, with 5 ppb having followed the lift-outs after an intermediate acid blank. Different sub-samples of the parent stock were used to produce either set of three. Despite this, there appears to be a pattern in the measurements: the first lift-out sample and the second bulk, the second lift-out and the first bulk, then the third sample from both are all at a similar difference to the assay value. The uncertainties, especially in the lift-outs, are very high so there is a good possibility that this is just a result of the pattern statistics. Alternatively, it could be possible that this is isotopic heterogeneity in the material that is hidden by ‘averaging’ in larger bulk samples: possibly this material is a blend of two or three different isotopic compositions (such as 1.6E-3 and 7E-3 ^235^U/^238^U) to achieve a target average ^235^U depletion. Further, more reliable, measurements are needed from either follow-up studies with the method improvements found here, or from other techniques such as nanoSIMS. The higher ratio for both samples had been discarded from the actual calculated values because of the high uncertainties and divergence from other values.

**Table 2:** ICP-QQQ-MS ^235^U/^238^U isotope ratios of lift-out and 5 ppb U samples calculated directly from counts-per-second of isotopes before any non-linearity or mass bias corrections, with standard uncertainties provided (*k* = 1). Sample numbers are in running order; there are no connections between lift-out and bulk samples

	Lift-out ^235^U/^238^U	Bulk 5 ppb ^235^U/^238^U
Sample 1	1.79(90)E-3	3.69(89)E-3
Sample 2	3.9(18)E-3	1.64(33)E-3
Sample 3	7.7(48)E-3	6.0(17)E-3

The isotope ratios, along with the UKNNL assay values for ^234^U and ^236^U, have been used to calculate the ^235^U weight percentage of the uranium in the UO_2_ material. These values are plotted in Fig. 4b alongside the TIMS measurements and assay value. The uncertainties in these ICP-QQQ-MS results are larger as a result of the propagation of uncertainties through the calculations. The lift-out result of 0.32 ± 0.12 wt% ^235^U resembles the TIMS lift-out 1 measurement of 0.3226 ± 0.0019 wt% ^235^U, and TIMS lift-out measurement average of 0.312 ± 0.006 wt% ^235^U, although the 37.5% relative combined standard uncertainty prevents much weight being given to it.

Despite the large measurement uncertainty, it is still possible to recognise that this material is depleted: if the expanded coverage of *k* = 2 is applied, the possible range of values remains below the natural ^235^U/^238^U value of 7.256 ± 0.004 × 10^− 3^ [22].

Although isotopic analysis was the primary goal of this work, elemental composition was also of interest in the ICP-QQQ-MS measurements and a wide-range of possible contaminants were examined. Unfortunately, acid contamination was too high to allow anything to be found in this material, highlighting the need for ultra-high purity acid in future trials. The improved method should produce higher uranium concentration solutions for analysis in the future, increasing the limit of detection for contaminants in the parent material itself. For specific trace elements of interest, isotope dilution could also be incorporated to improve quantification [23].

## Conclusion

Nanogram samples (between 2 and 80 ng) of uranium dioxide were extracted from a solid pellet using a gallium dual-beam FIB-SEM, then analysed by TIMS and ICP-QQQ-MS to measure the composition of major U isotopes. Using TIMS, three 2 ng lift-outs were measured for weight-percentages of the following isotopes with associated relative combined standard uncertainty (*k* = 1): ^234^U (5.6%), ^235^U (0.53%), ^236^U (11%), and ^238^U (0.26%). ICP-QQQ-MS was used to analyse 60 to 80 ng lift-outs for ^235^U with a 37.5% relative combined standard uncertainty.

Capabilities for the extraction, chemical preparation, and elemental separation of nanogram uranium samples, as well as their subsequent isotopic and elemental analysis were established. Methods were developed to solve challenges as they appeared and published techniques to improve detection limits and measurement certainty have been incorporated to improve future data acquisition. With the development of these capabilities, it will then be possible to probe the heterogeneity of bulk materials by selective sampling and analysis of points or phases of interest.

The next scheme of work will aim to use the improved methods on a larger scale by extracting a greater number of lift-outs from across a bulk material, tracking the location of each so that isotopic and elemental measurements can be related back to particular phases or areas. These data can then be combined and compared against bulk measurement techniques to determine the extent to which nano-scale sampling can provide macro, as well as micro, information.

## Data Availability

The data are available by the authors.
